# Investigating the Efficiency of Ultraviolet Photodissociation in Peptides Modified with *N*‑Terminal UV-Absorbing Chromophores

**DOI:** 10.1021/jasms.5c00389

**Published:** 2026-03-25

**Authors:** Nikita Levin, Yana Demyanenko, Eduardo Kitano, Shabaz Mohammed

**Affiliations:** † The Rosalind Franklin Institute, Harwell Science and Innovation Campus, Didcot OX11 0QX, U.K.; ‡ Department of Pharmacology, University of Oxford, Oxford OX1 3QT, U.K.; § Department of Biochemistry, University of Oxford, Oxford OX1 3QU, U.K.; ∥ Department of Chemistry, University of Oxford, Oxford OX1 3TA, U.K.

## Abstract

Ultraviolet photodissociation (UVPD) has emerged as a powerful alternative to conventional collision-induced dissociation (CID) for peptide and protein sequencing in mass spectrometry-based proteomics. However, the UVPD efficiency depends on the presence of UV-absorbing chromophores within analytes. Here, we systematically investigate how *N*-terminal modification of peptides with aromatic chromophores influences the 193 nm UVPD efficiency and fragmentation behavior. Using NHS ester chemistry, human cell lysate digests were derivatized with a range of UV-absorbing aromatic labels and analyzed on an Omnitrap–Orbitrap platform equipped with a 193 nm ArF excimer laser. Compared with unmodified controls, derivatized peptides displayed a modestly increased fragmentation efficiency and a greater diversity of sequence-informative fragments, particularly *b*-type ions. Peptides lacking aromatic amino acids benefited most from N-terminal aromatic labeling, exhibiting enhanced fragmentation and sequence coverage. These findings demonstrate that incorporation of aromatic chromophores at the N-terminus can enhance UVPD performance and improve spectral interpretability, thereby expanding the applicability of UVPD in bottom-up proteomics workflows.

## Graphical abstract

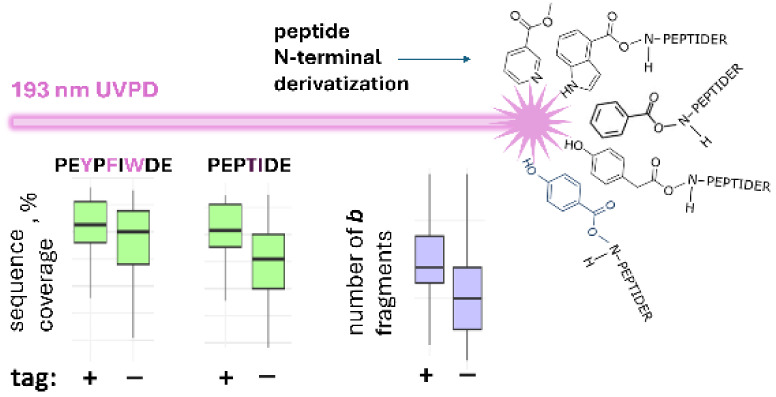

## Introduction

Gas-phase fragmentation of ionized peptides with subsequent detection of the product masses is the key step in sequence determination in mass spectrometry-based proteomics analysis. Collision-induced dissociation (CID) is by far the most popular method to fragment peptides due to its outstanding speed and efficiency.^1^ While perfectly suitable for most applications in bottom-up proteomics, CID often falls short in the analysis of intact proteins and protein modifications. Alternative methods have been developed, including the irradiation of analyte ions by electrons or laser light in a range of energies and wavelengths, respectively.^2^ Ultraviolet photodissociation (UVPD) is a well-characterized method for sequencing biomolecules^3,4^ offering information-rich spectra complementary to standard CID. Originally described by the groups of McLafferty, Russell, Kim, and Reilly who studied UVPD of peptides on custom time-of-flight (TOF) and Fourier transform ion cyclotron resonance (FT-ICR) mass spectrometers,^5–8^ UVPD was further developed and implemented for a broader range of biomolecules by Brodbelt and coworkers, who primarily employed a 193 nm laser within modified commercial ion trap instruments.^3,4^ Furthermore, the transition to high-resolution, high-mass accuracy instrumentation, through the initial use of orbitraps, has greatly improved the confidence of mass assignment in often highly congested UVPD spectra.

In a standard UVPD experiment, a cloud of precursor ions is irradiated with a beam of UV light, resulting in fragmentation. While the underlying mechanism is debated and may involve radical-driven and/or CID-like fragmentation pathways,^9^ the efficiency of UVPD is directly linked to the rate of resonant absorption of a UV photon by the analyzed molecule. The latter is dictated by the availability of UV-absorbing chromophores within the analyte and the power output of the laser. A variety of laser light wavelengths are available, including, but not limited to, 355, 266, and 213 nm (third, fourth, and fifth harmonics of Nd:YAG laser, respectively), 193 nm (ArF excimer laser), 157 nm (F_2_ excimer laser), and a range of tunable wavelengths available through optical parametric oscillators and synchrotrons. Organic molecules typically absorb better in the far-UV region, i.e., at shorter wavelengths, and a single pulse of a 157 nm laser was shown to be sufficient for efficient dissociation of peptides.^10,11^ The major limitation of irradiation at this wavelength is its significant absorbance by air, which requires a vacuum along the beam path. A close alternative is an excimer laser emitting at 193 nm. It produces less congested spectra and requires slightly higher pulse energies,^11^ but importantly, it is compatible with the atmosphere, which simplifies the design of a mass spectrometer. Other wavelengths have also been investigated, such as harmonics of conventional Nd:YAG lasers (355, 266, and 213 nm). The 213 nm light was shown to produce fragmentation very similar to 193 nm in UVPD of peptides^12^ and proteins,^13^ but the relatively low power of such lasers require longer irradiation times, which are incompatible with state-of-the-art workflows in bottom-up proteomics. Higher laser fluences can be achieved for lower harmonics (355 and 266 nm), but these wavelengths suffer from the lack of suitable chromophores in polypeptides and, in general, generate products very similar to those of CID.^14–20^

Various strategies targeting the physicochemical properties of analytes have been employed to improve the efficiency and reduce the spectral complexity of UVPD, including derivatization of primary amines,^15,16,20–25^ C-termini,^17,23^ histidine and tyrosine residues,^26^ and UV-absorbing cross-linkers^27,28^ within peptides. Aromatic compounds are known to enhance the absorption of mid-to-far UV light^19–21^ due to the favorable electronic transitions within their conjugated π-electron systems. Modifying peptide sequences with such compounds therefore leads to higher efficiencies of UVPD, in particular for peptides that do not contain aromatic amino acids (tyrosine, phenylalanine, tryptophan, and histidine),^20,21,25^ or improves the spectral cleanliness and predictability of N-term-modified LysN peptides (typically not containing basic amino acids at C-termini).^24^

Chemical derivatization of peptides has so far primarily been viewed as a means to improve UV absorption and, eventually, the efficiency of UV photodissociation. For example, the addition of an aromatic group to a peptide N-terminus greatly reduces the energy required for efficient photofragmentation by a 266 nm laser of peptides lacking aromatic amino acids,^19^ and a similar, although more modest, effect has been observed for 193 nm UVPD of N-terminally derivatized peptides.^21,25^ The effect of some chromophore labels on the yields of peptide fragments of various types in 193 nm UVPD of tryptic or LysN peptides has also been discussed.^21,24,25^ Although useful for understanding UVPD mechanisms and developing methods for UVPD of peptides, previous reports were limited to anecdotal evidence focusing on the photofragmentation of a few selected peptides. In this work, we employ NHS ester chemistry to study the 193 nm UVPD of peptides modified N-terminally with a broader range of aromatic chromophores, and we extract global photofragmentation patterns from total proteomics data acquired from cell digest LC-MS data. In particular, we discuss how the presence of a label affects the efficiency of fragmentation and the generation of C- and N-terminal ions in general, and we ask the question of whether the absence of any aromatic residue in peptides affects their photodissociation by 193 nm UV.

## Experimental Section

### Materials

[2,5-Dioxopyrrolidin-1-yl nicotinate], [2,5-Dioxopyrrolidin-1-yl 1H-indole-4-carboxylate], solvents, sodium dodecyl sulfate (SDS), sodium deoxycholate (SDC), triethylammonium bicarbonate (TEAB) buffer, Benzonase, 2-chloroacetamide (2-CAA), formaldehyde, cyanoborohydride, and ammonium bicarbonate (ABC) were purchased from Sigma-Aldrich (Gillingham, UK); tris­(2-carboxyethyl)­phosphine (TCEP) bond breaker was obtained from Thermo Fisher Scientific (Altrincham, UK); sequencing-grade trypsin was acquired from Promega (Chilworth, UK); ArgC was purchased from KPL ApS (Copenhagen, Denmark); [2,5-Dioxopyrrolidin-1-yl benzoate] and [2,5-Dioxopyrrolidin-1-yl 4-hydroxybenzoate] were obtained from Fluorochem (Hadfield, UK); [2,5-Dioxopyrrolidin-1-yl 2-(4-hydroxyphenyl)­acetate] was acquired from BLDpharm (Reinbek, Germany).

### Lysate Preparation with Dimethylation for Trypsin/ArgC Digestion

Expi 293F (Gibco) cell pellet was solubilized in lysis buffer (1% SDS, 50 mM TEAB) and either treated with 1 μL of Benzonase for 30 min at 37 °C or sonicated in a Bioruptor Pico (Diagenode) for 30 cycles (30 s on/30 s off). Extracted proteins were then reduced and alkylated using 10 mM TCEP and 50 mM 2-CAA for 30 min at room temperature. Free amines were dimethyl-labeled with 40 mM formaldehyde and 20 mM cyanoborohydride overnight at 25 °C, with shaking.^29^ After quenching the reaction mixture with 100 mM ABC, labeled proteins were precipitated by adding four volumes of methanol and one volume of chloroform, followed by three volumes of water. The resulting mixture was centrifuged at 9000 *g* at 4 °C for 5 min. The protein pellet was washed with methanol, resuspended in 1% SDS in 50 mM TEAB buffer, and digested using a modified SP3 protocol.^30^

### Trypsin/ArgC Digestion

Proteins solubilized in 1% SDS in 50 mM TEAB were precipitated onto a 1:1 mixture of carboxyl-coated Sera-Mag SpeedBeads (65152105050250, 45152105050250, Cytiva) by the addition of neat acetonitrile to an 80% final concentration and incubation at 37 °C for 15 min. The beads were then washed twice with 80% ethanol and twice with 100% acetonitrile before incubation with digestion buffer (50 mM TEAB containing a 1:25 ratio of ArgC or sequencing-grade trypsin) for 4 h at 37 °C. At the end of the digestion, the supernatant containing peptides was retained, the beads were washed once with 20 μL of 2% DMSO, and the resulting solution was combined with the supernatant.

### Peptide Labeling with Chromophores

Peptides in SP3 digestion buffer or desalted dry peptides resuspended in 50 mM TEAB were labeled with approximately 100 nmol of each label (in neat acetonitrile or DMSO) per μg of peptide for 2 h at room temperature. Labeled peptides were diluted 10x in 0.1% formic acid and desalted using Oasis HLB 2 mg 96-well plates (Waters, UK) according to manufacturer’s guidelines. Eluted peptides were diluted in the sample solvent (5% formic acid, 5% DMSO in water) for LC-MS/MS analysis.

### LC-MS/MS Analysis

LC-MS/MS data were acquired using an UltiMate 3000 nanoUHPLC system (Thermo Fisher Scientific) coupled to an Orbitrap Exploris 480 (Thermo Fisher Scientific) modified with an Omnitrap (Fasmatech).^31,32^ The Omnitrap was equipped with a 193 nm ArF excimer laser (Coherent) with a repetition rate of 200 Hz and a maximum pulse energy of 12 mJ. Peptides were trapped on a C18 PepMap100 precolumn (300 μm i.d. × 5 mm, 100 Å, Thermo Fisher Scientific) using solvent A (0.1% formic acid in water) and then separated on an in-house packed analytical column (50 μm i.d. × 50 cm, in-house packed with ReproSil Gold 120 C18, 1.9 μm, Dr. Maisch GmbH). The composition of solvent B (0.1% formic acid in acetonitrile) changed from 12% to 40% over 15 min. Full-scan MS1 spectra were acquired in the Orbitrap (scan range 350–1400 *m*/*z*, resolution 60000, AGC target 300%). The top 10 most abundant peptides were selected each round of DDA for fragmentation. UVPD was performed in the Omnitrap; its products were mass-analyzed in the Orbitrap at 30000 resolving power, the AGC value was set to 200%, and the maximum injection time was set to 50 ms.

### Data Analysis

Raw data were analyzed using FragPipe (v23.1) MSFragger 4.3 ^33^ with standard ″closed” search settings against the UniProt database (UPR_Homo sapiens_9606 accessed on 24.10.29). The number of missed cleavages was set to two when searching trypsin data (allowed cuts only after arginine residues) and to four when searching ArgC data. Lysine dimethylation and cysteine carbamidomethylation were set as fixed modifications. Methionine oxidation and N-terminal derivatization were set as variable modifications. Mass accuracy was specified as 20 ppm for fragment ions and 10 ppm for precursor ions. Data were searched using *b-* and *y*-ion series, following our recent findings in the study of nonmodified peptides.^34^ MSBooster and all of the quantification parameters were disabled. Results were reported at 1% protein-level FDR. Intensities of precursor ions and peptide main-series fragments were extracted using the IPSA web browser tool.^35^ Data postprocessing was performed using in-house developed R scripts (RStudio, build 387). Photodissociation efficiency (PD efficiency, also referred to as the efficiency of UVPD) was calculated as the ratio between the summed up intensities of fragments to the summed up intensities of fragments and remaining precursor ions (TIC – total ion current):

$$\mathrm{PD efficiency}=\frac{\mathrm{TIC}\left(\mathrm{fragments}\right)}{\mathrm{TIC}\left(\mathrm{fragments}+\mathrm{remaining precursor}\right)}$$

## Results and Discussion

Chemical labels containing aromatic functionalities have been shown to enhance the efficiency of UVPD, in particular for peptides lacking aromatic amino acids,^21,25^ and to redistribute the frequencies and abundances of C- and N-terminal types of UVPD-characteristic peptide fragments.^24,25^ To examine these effects, we analyzed human lysate digests that were modified with a range of compounds containing aromatic moieties. We chose compounds that were similar in appearance to phenylalanine, tyrosine, and tryptophan (Figure 1, Supplementary Figure S1). These residues have a broad absorption band below 200 nm,^36^ compatible with our 193 nm laser. We also opted to derivatize with a nicotinate, which would then be the only label to maintain a charge at the N-terminus after derivatization.

**Figure 1:**
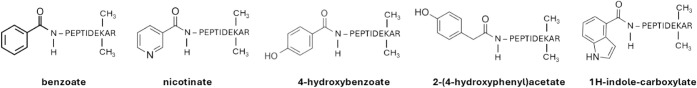
Generalized structures of N-terminally derivatized peptides with dimethylated lysine residues.

Our choice of amine-reactive chemistry led us to choose ArgC alongside trypsin for the digestion of human cell lysate. These NHS esters will block amines and reduce proton affinity,^22^ as well as decrease peptide polarity. Excessive use of these labels may result in the majority of tryptic and ArgC peptides having a charge state of +1, causing them to be ignored by the MS software during acquisition to prevent fragmentation events on nonpeptide ions. Furthermore, in a significant minority of cases, the peptides will be insoluble in the reverse-phase loading solvent. To ensure a charge state above +1, we blocked all lysine residues with dimethylation prior to digestion. This allowed the derivatization of peptides with aromatic tags placed solely at the N-terminus. The creation of dimethyl lysines causes trypsin to produce peptides similar to ArgC and ensures the digest contains a significant population of peptides possessing at least two charges. Limiting chemical derivatization by aromatic tags to only one site also helps avoid the crashing out of labeled peptides in aqueous solutions caused by excessively high hydrophobicity of peptides carrying more than one such label. Nevertheless, we still observed a slight drop in the peptide charge state distribution and increased peptide retention (Supplementary Figure S2). After a quick screening, we chose peptides labeled with nicotinate, benzoate, and non-NHS-labeled (control) to calculate the optimal number of pulses at a fixed energy of 6 mJ per pulse. Due to the lower number of identified modified peptides compared to nonmodified peptides, incurred by greater sample losses during the extended sample preparation, incomplete derivatization, and changed hydrophobicity (Supplementary Figure S2), we focused on the relative fragmentation performance caused by the NHS-labeling and therefore reported PSMs normalized to the maximum number in a series. As we stepped up the number of pulses, we initially observed poorer identification of modified peptides compared to nonmodified peptides (Figure 2A). However, the identification of modified peptides improved more rapidly as we increased the number of pulses than the nonmodified peptides, leading to all peptides requiring the same number of pulses for sufficient fragmentation. We found that four laser pulses were optimal in the LC-MS analysis of all three samples when *b-* and *y*-types of fragments were used for analysis. The same parameters were previously found to be optimal for nonlabeled, nondimethylated tryptic peptides.^34^ Using these parameters, we remeasured all of the ArgC and trypsin samples. To assess the fragmentation efficiency on the spectral level, we limited our data analysis to doubly and triply charged precursors and calculated the total ion currents (TIC) of main-series peptide fragments and remaining parent ions using the IPSA web browser tool.^35^ When comparing unique sequence-charge state pairs present in both labeled and control pools, we found that labeled peptides were fragmented slightly more efficiently in derivatized ArgC and trypsin digests than their counterparts in control ArgC and tryptic digests (Figure 2B,C).

**Figure 2:**
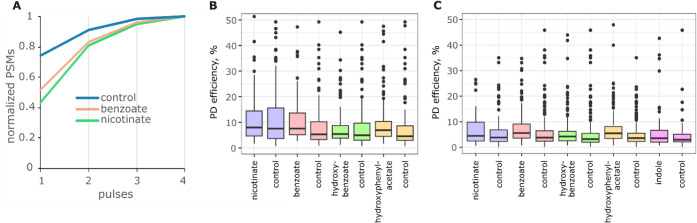
A) Normalized numbers of PSMs identified in the ArgC samples of nonderivatized (control) peptides and peptides derivatized N-terminally with nicotinic or benzoic acids, plotted against the number of UV laser pulses; *b-*,*y*-fragment types were used for identification. In the analysis of the derivatized samples, all nonlabeled precursors were discarded. B,C) Efficiency of UVPD in ArgC (B) and trypsin (C) samples. Doubly and triply charged precursors were selected for analysis. Each control contains the same sequences and charge states as the corresponding labeled sample (like-to-like comparison).

Initial inspection of spectra corresponding to the UVPD of N-terminally derivatized peptides with the same sequence suggested a subtle effect caused by labeling (Figure 3, Supplementary Figure S3). The analysis revealed that while the efficiency of UVPD is similar in labeled peptides compared to the control, the presence of an aromatic tag at the N-terminus of the peptide not only marginally improved the signal for the same fragments but also increased the number of observed fragments. The formation of *b*_1_ in labeled peptides (Figure 3B–F) can be explained by the addition of the carbonyl group.^37^

**Figure 3:**
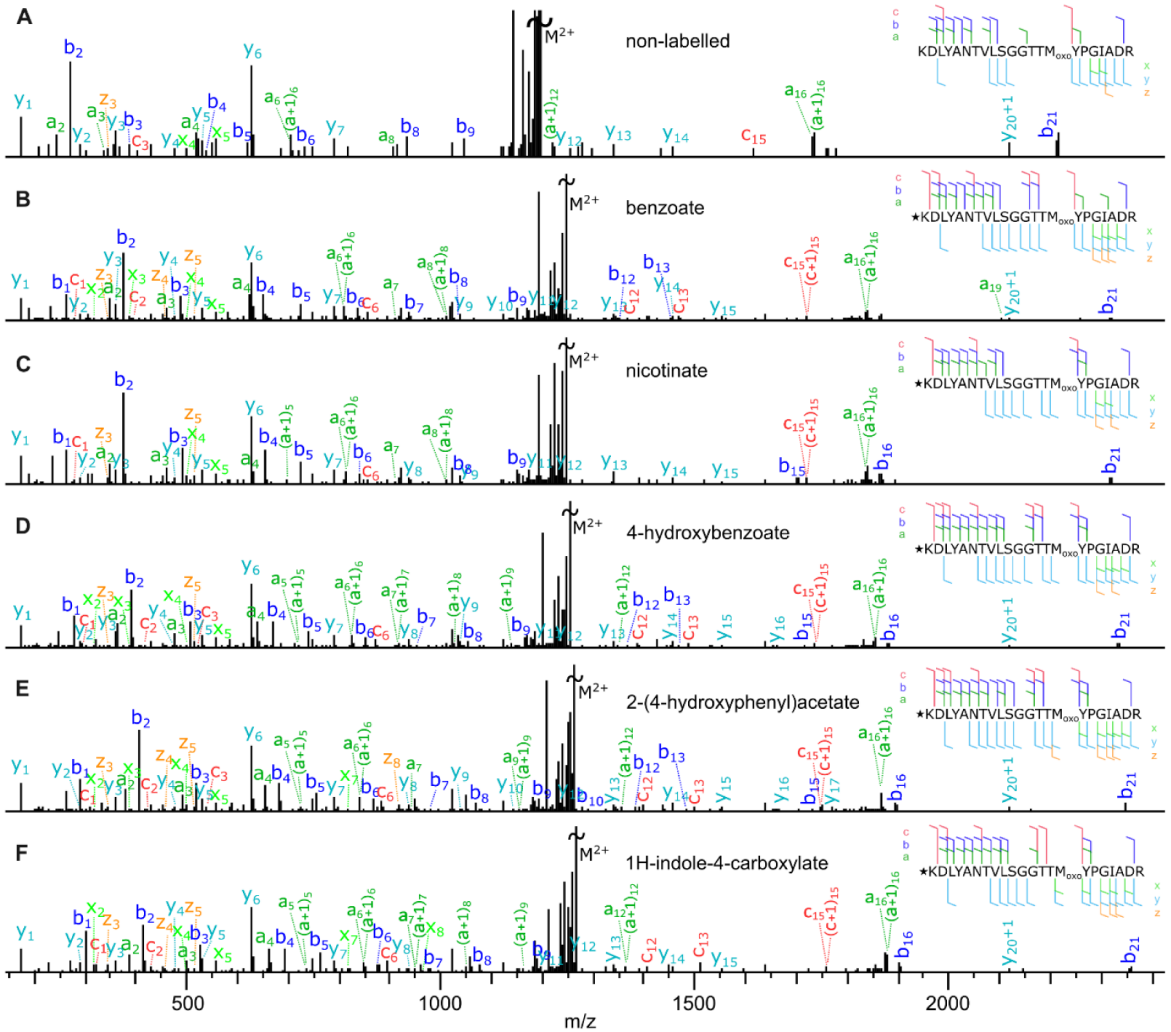
MS/MS UVPD spectra of the doubly charged KDLYANTVLSGGTTM­(Oxo)­YPGIADR peptide A) and of the same peptide N-terminally modified with benzoate B), nicotinate C), 4-hydroxybenzoate D), 2-(4-hydroxyphenyl)­acetate E), and 1H-indole-4-carboxylate F). The star symbol ★ marks the N-terminal modification. The lysine residue is dimethylated. Supplementary Figure S3 shows Panels A and **E** zoomed in to the 150–1000 *m*/*z* range.

In a subsequent data analysis, we tried to answer the question of how aromatic labels placed at the N-terminus can affect the population of UVPD-characteristic fragments in a broader range of peptides. We collated the main-series fragment-ion information for all the data and generated plots both in terms of frequency of observation and relative intensity for each fragment type in peptides with and without aromatic amino acids (Figure 4A,B, Supplementary Figure S4A,B). Similarly to what has been previously reported for UVPD of tryptic peptides,^12,38,39^ *b-* and *y*-fragments dominate UVPD spectra of lysine-dimethylated ArgC and tryptic peptides (i.e., peptides containing a C-terminal arginine and occasionally an internal dimethylated lysine(s)). Notably, the presence of a chemical tag significantly increases the numbers of *b*-type ions for all labels, especially in the peptides lacking aromatic amino acids (Supplementary Figure S5). As somewhat expected, nicotinate peptides possessed the most significant difference in the number of *b*-ions between labeled and control groups in ArgC samples (the trypsin experiment had too few numbers for a statistical analysis, Supplementary Figure S2B). It is noteworthy that the peptide charge distribution for the nicotinate labeling experiment was similar to the other labels (Supplementary Figure S2D). We reasoned that the nicotinate *pK*_*a*_ ∼ 5 was insufficient to replicate the behavior of an unmodified N-terminus, which can have a *pK_a_* ∼ 8. The frequencies of *y*-ions were also marginally higher in the UVPD of labeled peptides, although their contribution to the total ion current (TIC) of fragments dropped over the range of labels (Figure 4A,B, Supplementary Figure S4A,B). Interestingly, while the average numbers of *c*-type ions were approximately the same in labeled and unlabeled precursors, their contribution to fragment TIC was substantially lower in labeled peptides without aromatic amino acids (comparing Figure 4A and B). The drop in intensities of *c*-ions, which are known to be products of radical-driven processes, can potentially be attributed to the radical-trapping nature of the label. Another interesting observation is the dependence of the ratios between the average frequencies of *b*:*y* and *b*:*a* ions (Supplementary Figure S6). While for *b*:*y* ions, this ratio depends on the presence of an N-terminal tag but is not sensitive to aromatic residues within a peptide, the production of *a-* ions benefits from an N-terminal tag but is clearly favored by the absence of any aromatics within the peptide backbone, potentially pointing to the interplay between mechanisms of formation of *b-* and *a*-ions in UVPD.

**Figure 4:**
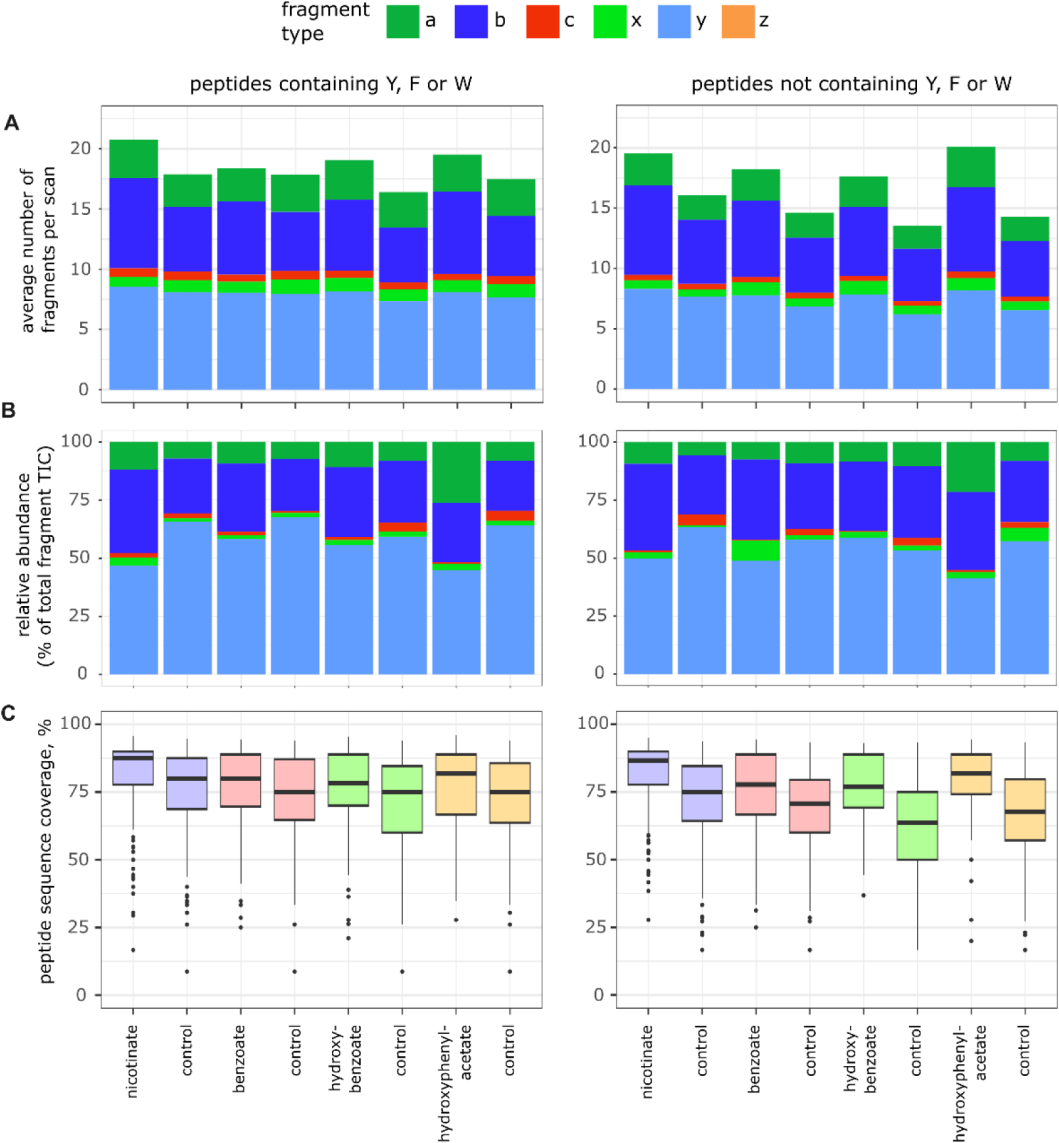
A) Total number of fragments of each type across all scans divided by the number of scans. B) Total intensities of fragments of each type across all scans divided by the overall intensity across all scans. C) Boxplot of peptide sequence coverages defined as the number of dissociated bonds normalized to peptide length. In all panels, doubly and triply charged precursors in ArgC samples that do (left) or do not (right) contain at least one aromatic amino acid residue (Y, F, and W) were selected for analysis. Each control contains the same sequences and charge states as the corresponding labeled sample (like-to-like comparison).

The labeling appears to increase the number of fragment ions generated without substantial improvements in fragment intensities (Figure 4A), rather than producing the same number of fragments but with higher intensity when compared to unlabeled peptides. An increase in the number of fragments will lead to signal splitting and a lower signal-to-noise for each fragment. This wider gamut of fragmentation and signal splitting helps rationalize why the identification rate of labeled peptides drops more quickly than that of unlabeled peptides for a suboptimal number of laser pulses (Figure 2 B,C).

Further, we investigated how this increased diversity of formed fragments translates into sequence coverages of analyzed peptides. We calculated peptide sequence coverage as the ratio between the number of dissociated bonds and the length of a peptide, counting a bond as dissociated if any of the possible fragment types (*a, b, c, x, y, z*) were detected at the corresponding cleavage site. Figure 4C and Supplementary Figure S4C show that UVPD of labeled peptides dissociates, on average, more bonds than UVPD of the same but nonlabeled precursors. We expected to see these improved sequence coverages reflected in higher scores assigned by the search engine. Supplementary Figures S7,S8 show hyperscores generated by MSFragger for labeled precursors plotted against the same but nonlabeled precursors, with all main-series fragment types used for analysis. Surprisingly, the hyperscores were distributed evenly on both sides of the plot separated by the identity line, with the only apparent exception being hydroxybenzoate. Increased signal of a fragment or addition of a few new fragments does not appear to affect the score significantly, which probably speaks to the already rich spectra generated by UVPD.

We then further narrowed our focus to peptides that do or do not contain aromatic amino residues, namely tyrosine (Y), phenylalanine (F), and tryptophan (W). The proportion of peptide sequences not containing at least one of these amino acids was notably lower (up to ∼15%) among sequences found in control samples, as opposed to sequences modified with a chromophore tag (Figure 5). These observations point out the bias of UVPD against peptides lacking aromatic residues and suggest a potential way to rectify this bias using chemical tags that mimic aromatic amino acids. An insight into fragmentation mechanisms would be greatly appreciated but is challenging to achieve. The primary aspect of UVPD gas-phase chemistry is absorbance, which is clearly favored by aromatics, as stated above. A 193 nm photon takes the system to a highly excited state, from which the system can emit a photon, transfer excitation energy to molecules of buffer gas via collisions, undergo nonradiative relaxation through multiple pathways, or dissociate. In the latter case, as hypothesized by Julian,^9^ the ion either undergoes internal conversion into excited vibrational modes followed by CID-like dissociation to produce primarily *b-* and *y*-fragments (given the vibrational energy is sufficiently high), or the excitation state couples to a dissociative state with subsequent fragmentation via an unknown pathway. Additional analytical methods, such as absorption or action spectroscopy, isotopic labeling, and computational methods, may be required to elucidate the structures of UVPD products and clarify reaction mechanisms. Our results (Figure 4, Supplementary Figure S4) show that the formation of *b*-ions seems to be promoted in UVPD of labeled species, arguably pointing to a preferential CID-like pathway following the absorption of a photon by aromatics. We point out, however, that these results should be considered and interpreted only in the context of the 193 nm UVPD of ArgC (and perhaps tryptic) peptides. Earlier studies showed that the location of charge is important, as demonstrated in UVPD of fixed-charge peptides^23^ or in UVPD of N-terminally imidazolinylated LysN peptides that show a strong series of *a*-, *b*-, and *c*-type ions and a lack of *x*-, *y*-, and *z*-ones.^24^ The wavelength and type of chemical tag are consequential too; for example, N-terminal derivatization by Alexa Fluor 350 or AMCA fluorophore completely precludes the formation of N-terminal fragments in 355 nm UVPD,^16^ and the same effect was reported for N-terminal derivatization by the SITS tag in 266 nm UVPD.^15^ The energetics of ion transfer within a reaction chamber is equally crucial, as shown in our recent large-scale LCMS study of nonlabeled peptides.^34^ We therefore conclude that different designs of mass spectrometers may have different yields of UVPD fragments.

**Figure 5:**
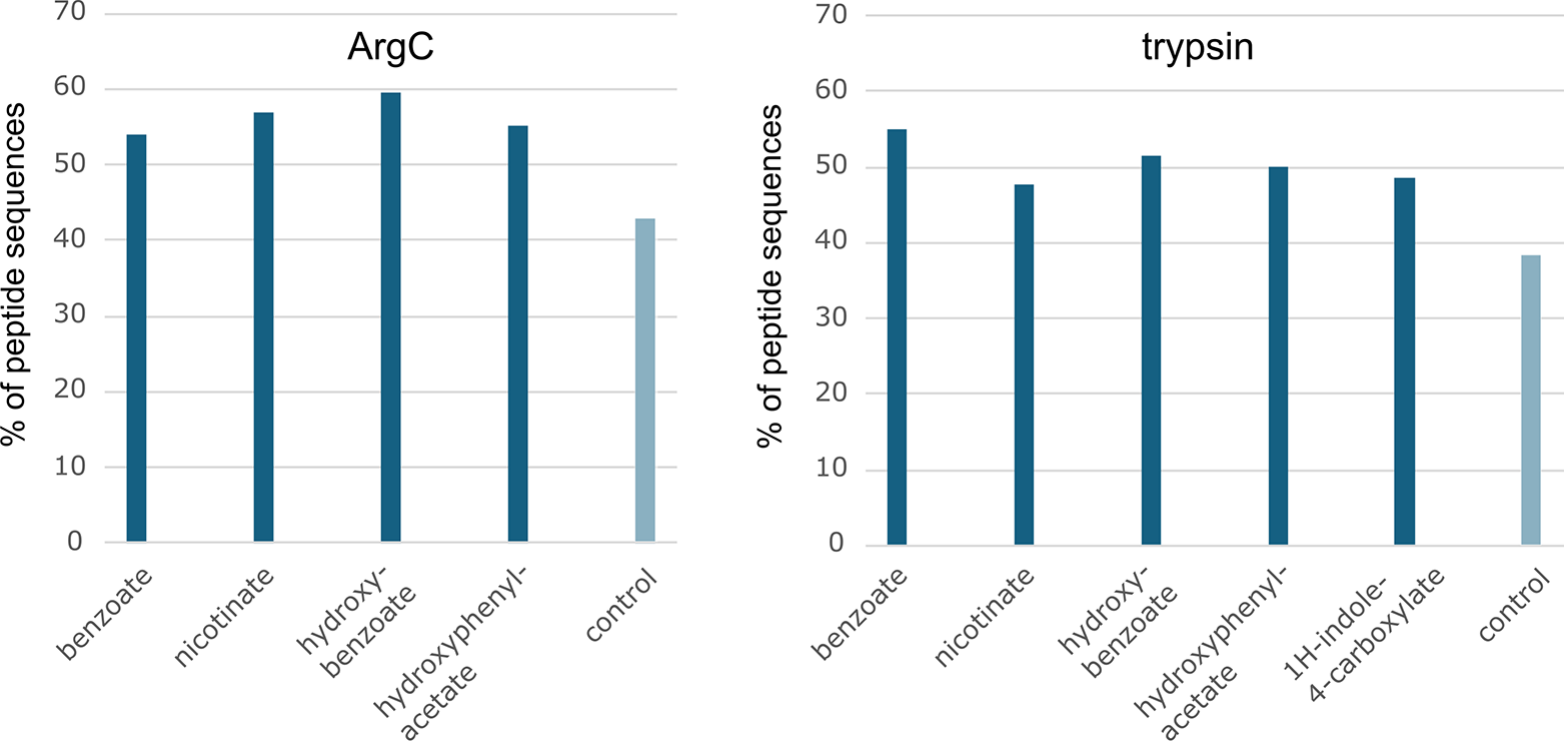
Percent of unique peptide sequences found in ArgC (left) and trypsin (right) samples that do not contain either Y, F, or W (at least one residue).

## Conclusion

UVPD is a potent technique in proteomics analysis, producing a large variety of sequencing fragments that make it perfectly suitable for diverse applications as complex as *de novo* sequencing of peptides. We and others have previously shown that the quality of data generated by UVPD in a typical bottom-up LCMS experiment is on par with state-of-the-art workflows employing collisional dissociation. The data produced in this work suggest that UVPD may be biased toward peptides containing aromatic amino acids. In view of this, we investigated the 193 nm UVPD of peptides N-terminally derivatized by chemical tags containing aromatic moieties. We discussed the numbers and intensities of peptide main-series fragments produced by UVPD and how they affect the quality of peptide sequencing. The results agree with earlier findings and expand the arsenal of known chemistries to a wider range of labels introduced by NHS-ester chemistry. This strategy can be employed to improve the interpretability and quality of UVPD spectra, perhaps accelerating the implementation of UVPD in bottom-up proteomics workflows.

## Supplementary Material



## Data Availability

The mass spectrometry proteomics data have been deposited to the ProteomeXchange Consortium via the PRIDE^40^ partner repository with the data set identifier PXD075227.

## Notes

NHS esters used for peptide N-terminal derivatization (Figure S1); total numbers of identifications, distributions of charge states, and retention times in derivatized and nonderivatized samples (Figure S2); zoomed-in areas of spectra from Figure 3A,E (Figure S3); efficiency of UVPD in trypsin samples (Figure S4); distributions of *b*-type ions in labeled vs nonlabeled peptides (Figure S5); ratios of average frequencies of *b*-type fragments to *y*-type and *a*-type fragments (Figure S6); distributions of hyperscores in control and labeled samples (Figures S7, S8) (PDF)
