# Supplementary material for: Investigating the Efficiency of Ultraviolet Photodissociation in Peptides Modified with N‑Terminal UV-Absorbing Chromophores
Source: J Am Soc Mass Spectrom. 2026 Mar 25;37(5):1124–31. doi: 10.1021/jasms.5c00389 (PMC13154361; doi:10.1021/jasms.5c00389)
Supplement: Supplementary file 1 [file js5c00389_si_001.pdf]

Supporting information

Investigating the efficiency of ultraviolet photodissociation of peptides modified with N-terminal UV-absorbing chromophores.

Nikita Levin[1,2], Yana Demyanenko[1,2], Eduardo Kitano[1,2], Shabaz Mohammed[1,3,4,*]

[1] The Rosalind Franklin Institute, Harwell Science and Innovation Campus, OX11 0QX, Didcot, UK

[2] Department of Pharmacology, University of Oxford, OX1 3QT, Oxford, UK

[3] Department of Biochemistry, University of Oxford, OX1 3QU, Oxford, UK

[4] Department of Chemistry, University of Oxford, OX1 3TA, Oxford, UK

*email: shabaz.mohammed@chem.ox.ac.uk

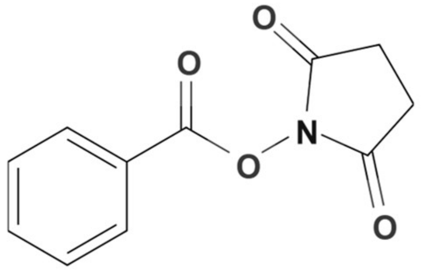

2,5-Dioxopyrrolidin-1-yl benzoate

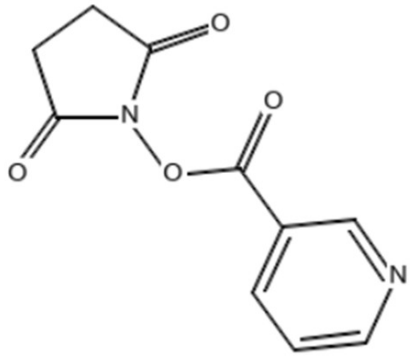

2,5-Dioxopyrrolidin-1-yl nicotinate

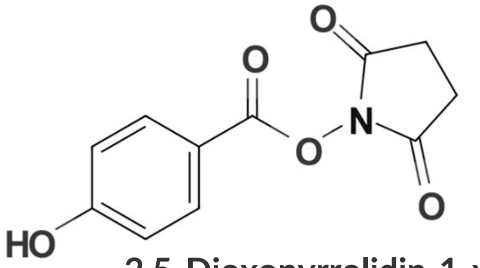

2,5-Dioxopyrrolidin-1-yl
4-hydroxybenzoate

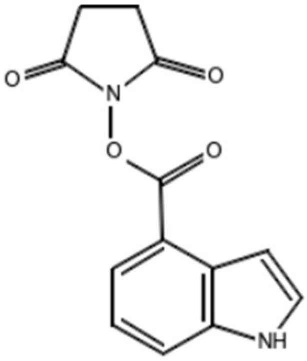

2,5-dioxopyrrolidin-1-yl
1H-indole-4-carboxylate

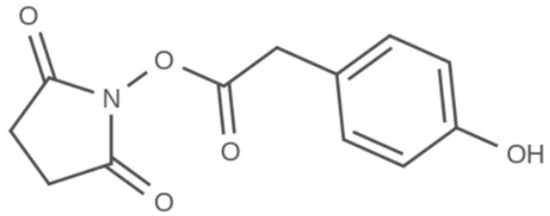

2,5-Dioxopyrrolidin-1-yl
2-(4-hydroxyphenyl)acetate

Supplementary Figure S1. NHS esters used to derivatize N-termini of peptides.

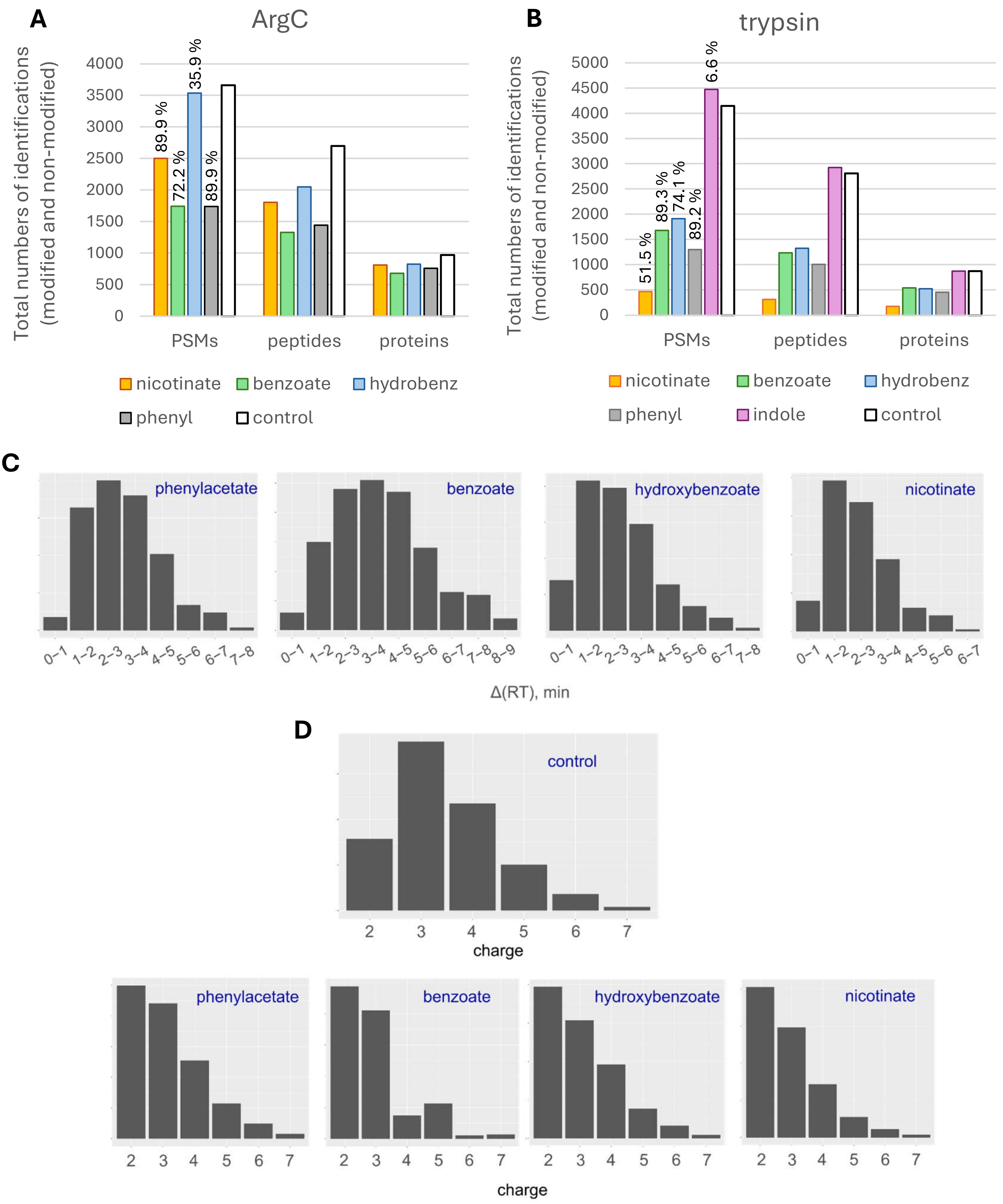

Supplementary Figure S2. A-B: Absolute numbers of identifications in derivatized and non-derivatized ArgC (**A**) and trypsin (**B**) samples. Percent number shows the proportion of PSMs identified as labelled by an aromatic tag. **C**: Binned distributions of differences between retention times of precursors in each derivatized sample and retention times of the same precursors in the control sample. **D**: Binned distributions of charge states of all precursors. Histograms in **C** and **D** were plotted for each combination of peptide sequence, non-aromatic-tag modification (e.g. methionine oxidation) and charge state in ArgC samples. All duplicates of such combinations were filtered for highest hyperscore.

KDLYANTVLSGGTTM(Oxo)YPGIADR

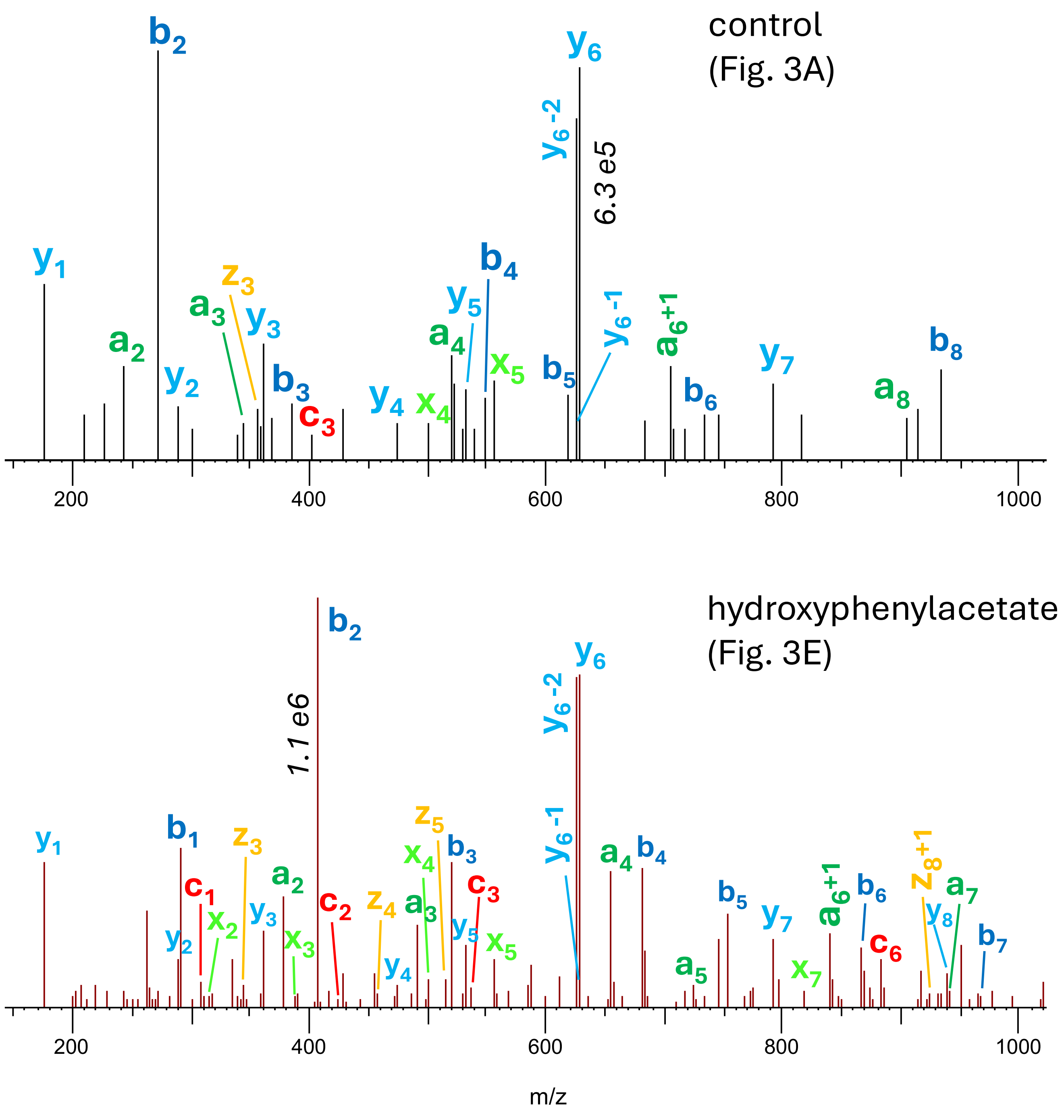

Supplementary Figure S3. Zoomed-in areas of spectra from Figure 3A (top) and Figure 3E (bottom).

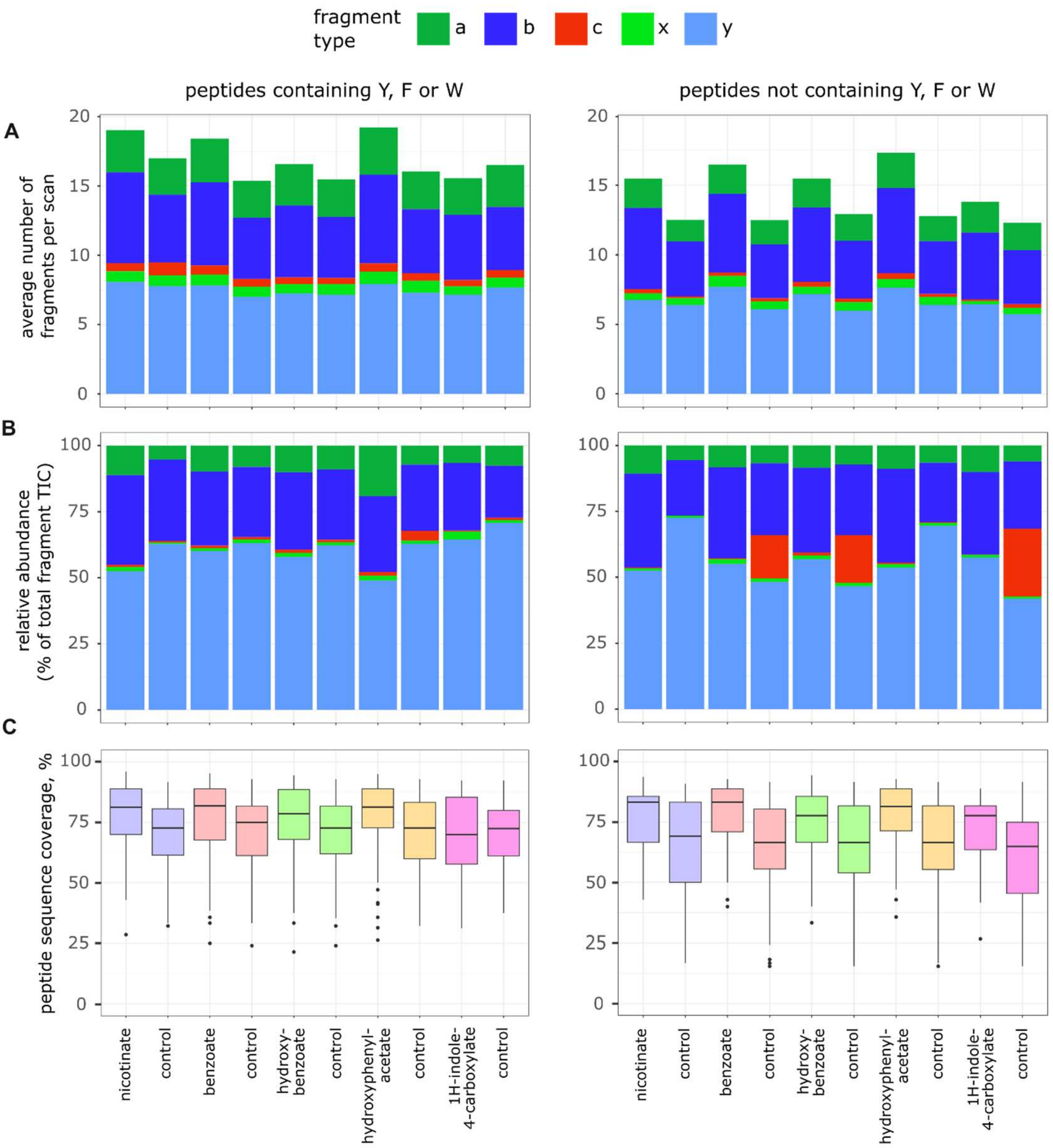

Supplementary Figure S4. A) Total number of fragments of each type across all scans divided by the number of scans. B) Total intensities of fragments of each type across all scans divided by the overall intensity across all scans. C) Boxplot of peptide sequence coverages defined as the number of dissociated bonds normalized to peptide length. In all panels, doubly and triply charged precursors in trypsin samples that do (left) or do not (right) contain at least one aromatic amino acid residue (Y, F, W) were selected for analysis. Each control contains the same sequences and charge states as the corresponding labelled sample (like-to-like comparison).

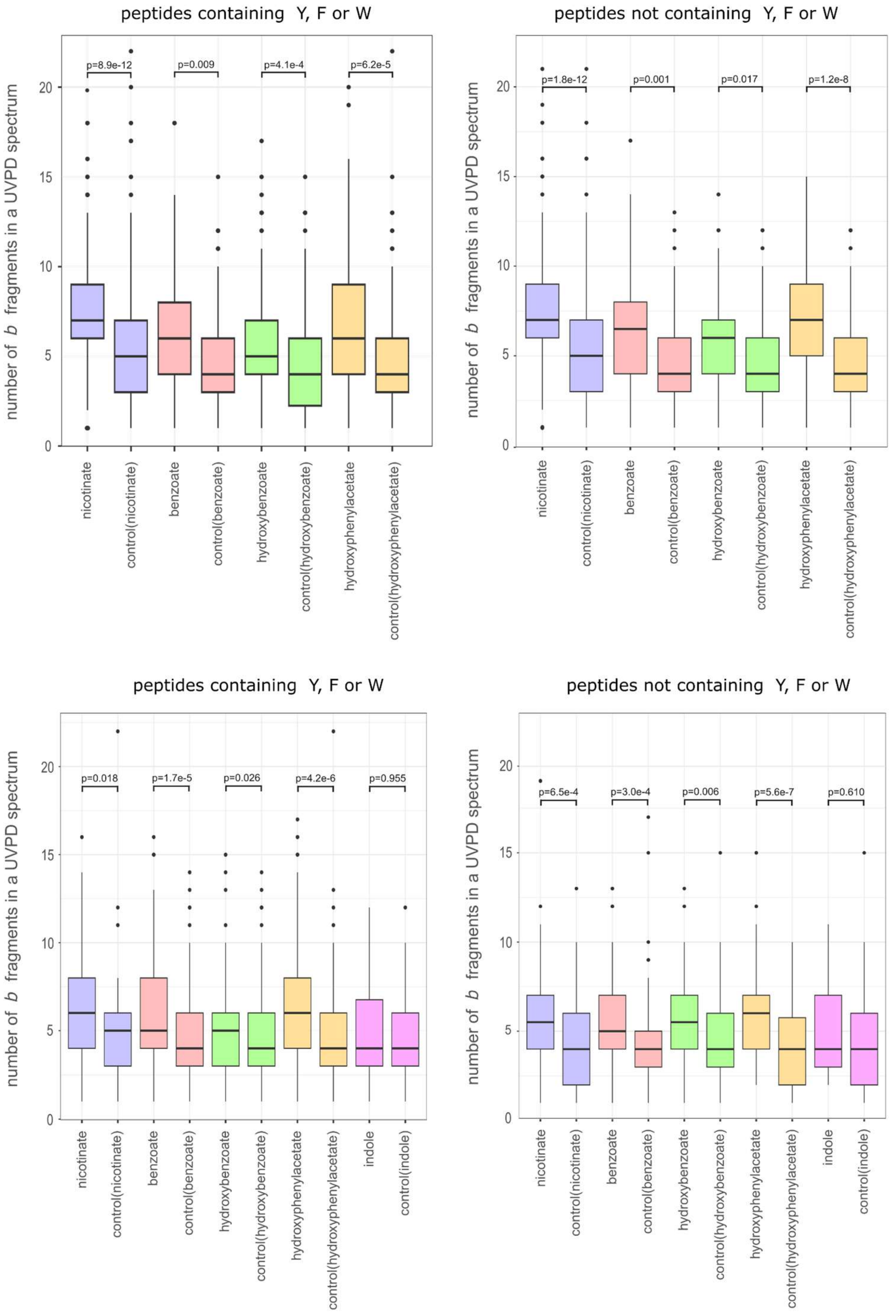

Supplementary Figure S5. Box plot distributions of numbers of *b*-type ions found in spectra of labelled peptides vs control in ArgC (top) and trypsin (bottom) samples. In all panels, doubly and triply charged precursors containing (left) and not containing (right) aromatic amino acids were selected for analysis. Each control contains the same sequences and charge states as the corresponding labelled sample (like-to-like comparison). *p*-values are derived from Welch's two-sample t-test.

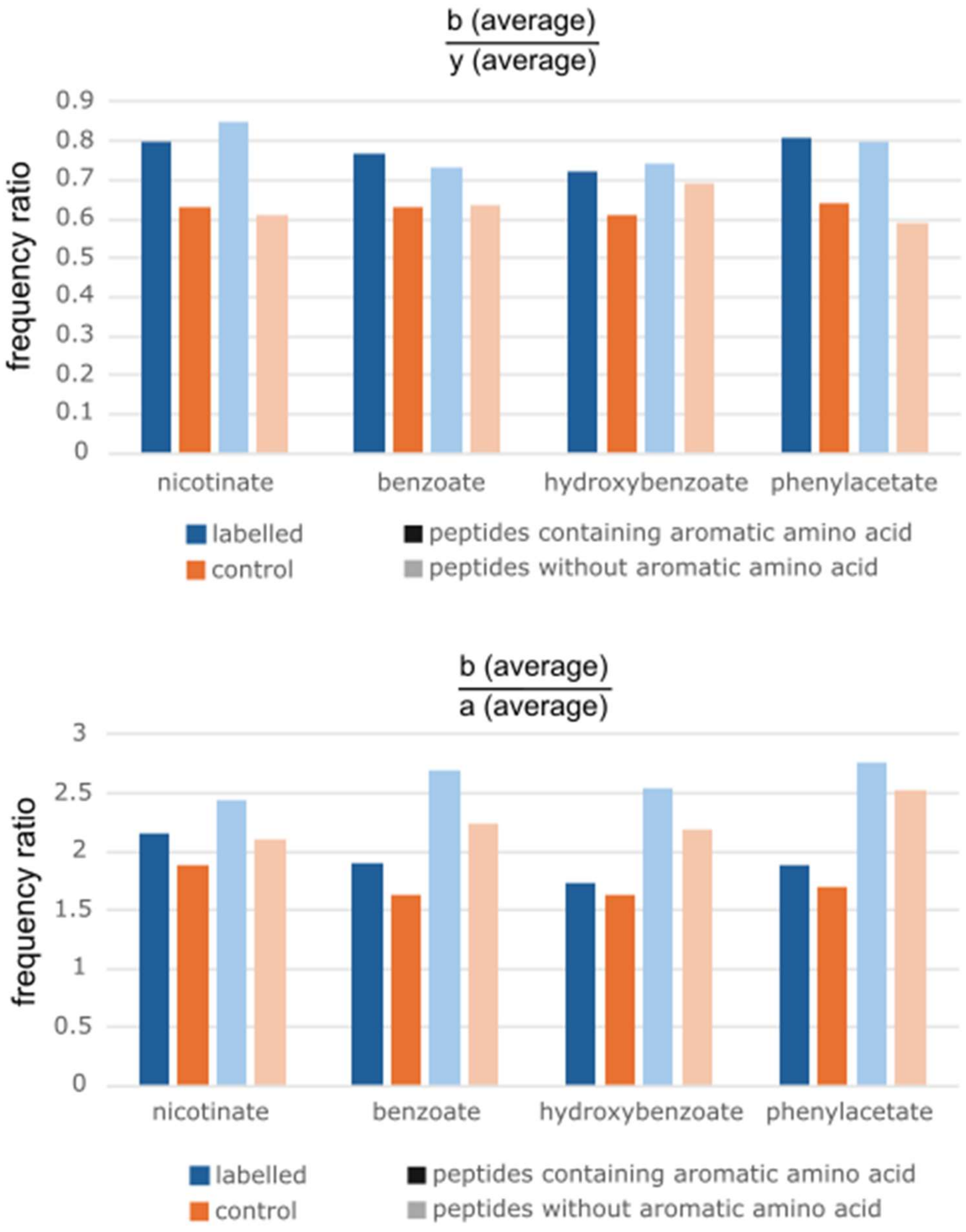

Supplementary Figure S6. Bar plots showing ratios of average frequencies of *b*-type fragments to *y*-type (top) and *a*-type (bottom) fragments in doubly and triply charged ArgC peptides containing and non-containing aromatic amino acids in labelled and non-labelled samples.

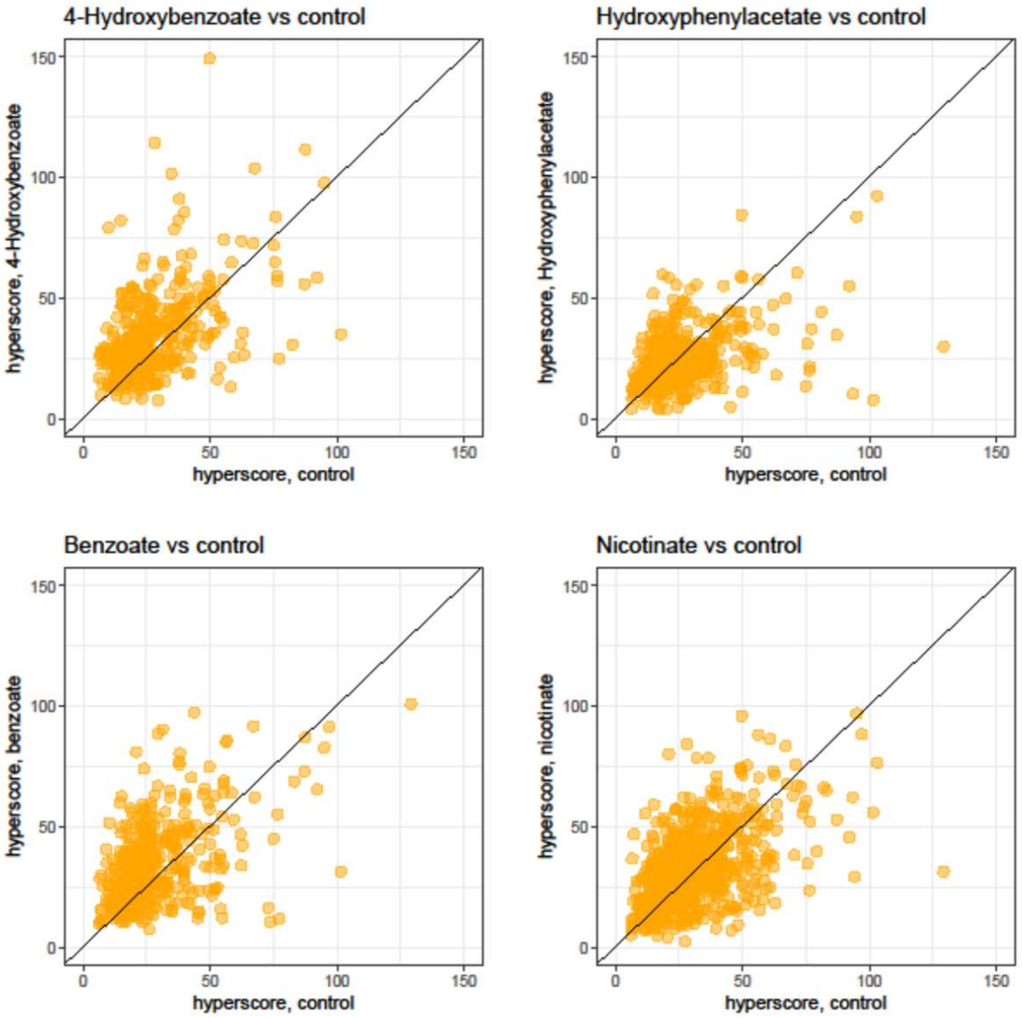

Supplementary Figure S7. Hyperscores generated by MSFragger for doubly and triply charged precursors in labelled ArgC samples plotted against the same precursors (same sequence and charge state) found in the control (non-labelled) sample. All main-series fragment types were used for analysis.

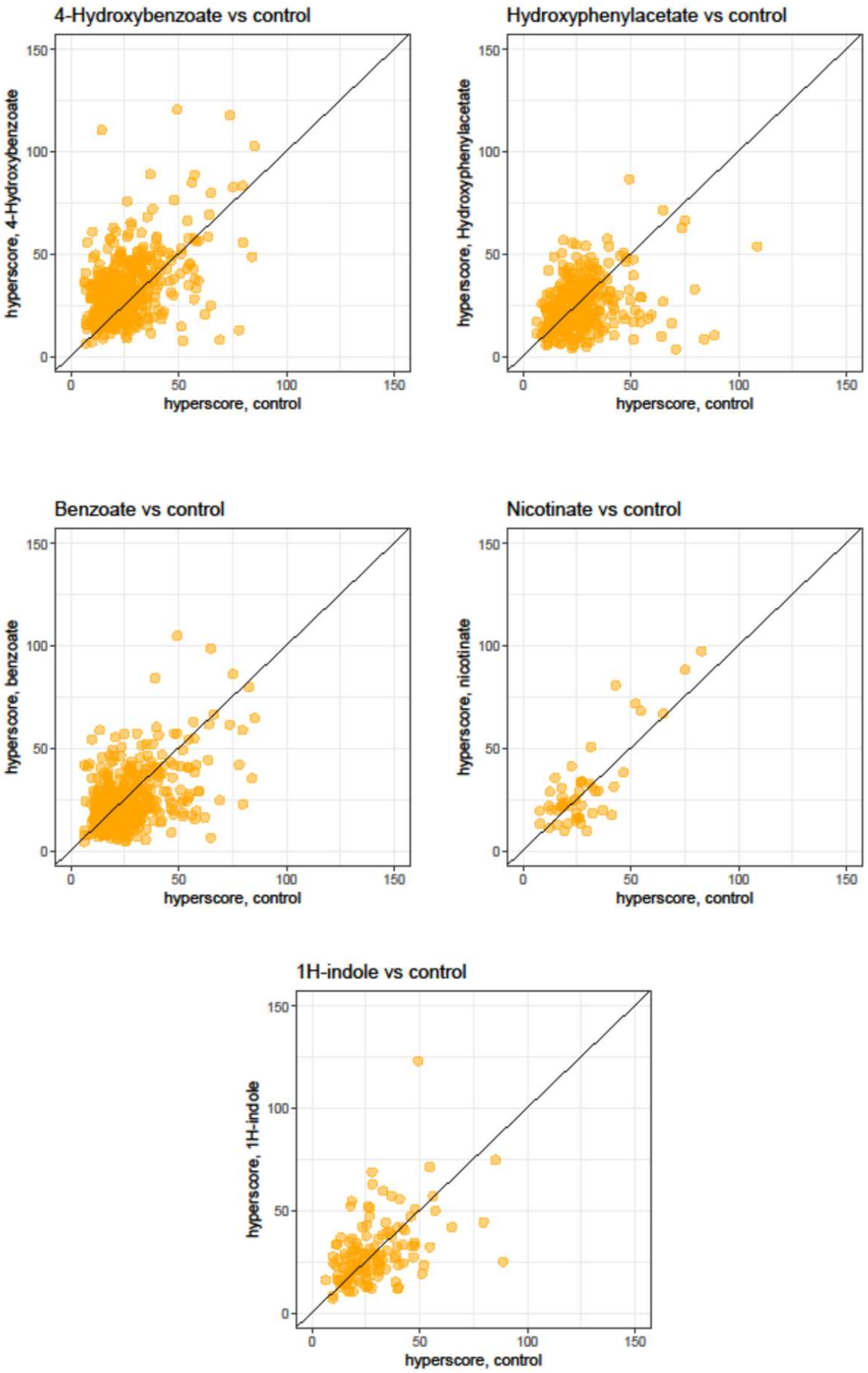

Supplementary Figure S8. Hyperscores generated by MSFragger for doubly and triply charged precursors in labelled trypsin samples plotted against the same precursors (same sequence and charge state) found in the control (non-labelled) sample. All main-series fragment types were used for analysis.
